# Double-edged sword: γδ T cells in mucosal homeostasis and disease

**DOI:** 10.1038/s12276-023-00985-3

**Published:** 2023-09-11

**Authors:** In Kang, Yumin Kim, Heung Kyu Lee

**Affiliations:** 1 Graduate School of Medical Science and Engineering, Korea Advanced Institute of Science and Technology (KAIST), Daejeon, 34141 Republic of Korea; 2 Department of Biological Sciences, KAIST, Daejeon, 34141 Republic of Korea

**Keywords:** Innate immune cells, Mucosal immunology, Innate immunity

## Abstract

The mucosa is a tissue that covers numerous body surfaces, including the respiratory tract, digestive tract, eye, and urogenital tract. Mucosa is in direct contact with pathogens, and γδ T cells perform various roles in the tissue. γδ T cells efficiently defend the mucosa from various pathogens, such as viruses, bacteria, and fungi. In addition, γδ T cells are necessary for the maintenance of homeostasis because they select specific organisms in the microbiota and perform immunoregulatory functions. Furthermore, γδ T cells directly facilitate pregnancy by producing growth factors. However, γδ T cells can also play detrimental roles in mucosal health by amplifying inflammation, thereby worsening allergic responses. Moreover, these cells can act as major players in autoimmune diseases. Despite their robust roles in the mucosa, the application of γδ T cells in clinical practice is lacking because of factors such as gaps between mice and human cells, insufficient knowledge of the target of γδ T cells, and the small population of γδ T cells. However, γδ T cells may be attractive targets for clinical use due to their effector functions and low risk of inducing graft-versus-host disease. Therefore, robust research on γδ T cells is required to understand the crucial features of these cells and apply these knowledges to clinical practices.

## Immune cells: Guardians of mucous membranes

A small population of highly active immune cells known as γδ T cells protect mucosal tissue, which lines interior body parts that contact the exterior (such as lungs, mouth, and stomach) against pathogens, but can also cause diseases. Mucosal surfaces, with their high pathogen exposure, require especially good immune protection. Heung Kyu Lee and co-workers at the Korea Advanced Institute of Science and Technology, Daejeon, South Korea, have reviewed the helpful and harmful effects of these little-studied cells. These γδ T cells guard against viruses, bacteria, and fungi, help determine which microbes inhabit our bodies, and even play roles in pregnancy. On the other hand, they can be involved in autoimmune diseases. Their ease of transplantation and high activity make them promising candidates for clinical applications, and trials in cancer treatment are underway.

## Introduction

The mucosa is a tissue that covers multiple body surfaces, including the respiratory tract, digestive tract, eye, inner ear, and urogenital tract. In addition to the skin, the mucosal surface is the boundary between the outside and the inside of the body. However, unlike the skin, which is protected by thick and cornified cell layers called the stratum corneum^1^, mucosal surfaces do not have a strong physical barrier. Because the mucosa has a high probability of contact with pathogens, mucosal surfaces are protected by cellular and noncellular immune systems. For example, most mucosal layers are covered by mucus, which prevents pathogens from contacting the mucosal epithelium^2^. The mucus layer includes many antimicrobial peptides, which prevent colonization by bacteria^3^. Various innate and adaptive immune cells suppress pathogens both on the luminal surface^4^ of the mucosa and in lymph node drainage^5^. γδ T cells play important roles in the cellular and noncellular immune systems. Because of their unique γδ T-cell receptor (TCR) usage, γδ T cells localize in the mucosa^6,7^ and directly and indirectly protect against pathogens. While αβ T cells recognize peptides processed and presented by the major histocompatibility complex (MHC), γδ T cells recognize other molecules, such as CD1d^8^, butyrophilins^9^, Skint1^10^, Annexin A2^11^, and EphA2^12^, which are associated with pathogenic infections and tissue damage. Because of their innate target recognition properties, γδ T cells can patrol the periphery that is not covered by T cells or B cells^13^. Although studies have revealed that the presence of γδ T cells is necessary to maintain homeostatic status^14^, these cells can also play a major role in the disruption of homeostasis^15^. In this review, we will discuss both the protective and disruptive roles of γδ T cells in mucosal homeostasis and diseases.

## Localization of γδ T cells in the mucosa

### Tissue specificity

γδ T cells are found in different organs, and their population in an organ is as heterogeneous as their TCR usage and functionality. Murine γδ T cells colonize specific organs based on their Vγ usage^16^. Generally, embryonic Vγ6+ γδ T cells are localized in the lung, gingiva, and reproductive tract; Vγ4+ γδ T cells are localized in the pulmonary tract and lung; and perinatally developed Vγ7+ γδ T cells are localized in the gut. The localization of Vγ7+ γδ T cells in the gut is influenced by the BTNL1-BTNL6 heterodimer which is expressed in the gut epithelium (Tonegawa nomenclature)^9,16^. In addition, in the urogenital tract, γδTCR+ cells expressing Vγ4 and Vδ1 are present in the mouse vagina and express CD5, CD28, CD25, and PGP-1^17^. Several factors affect the localization of γδ T cells to specific organs. In the case of gut-localized γδ T cells, surface molecules affect their homing to the intestine. In mice, intestinal epithelial cells express BTNL1 and BTNL6, which form heterodimers and stimulate Vγ7+ γδTCRs, thereby inducing the selection and residence of Vγ7+ γδ T cells in the gut^9^. In addition to BTNL1-BTNL6-mediated expansion, Vγ7+ γδ T cells express a greater amount of integrin α4β7, a binding partner of MAdCAM1, than conventional T cells, which facilitates the localization of Vγ7+ γδ T cells in the gut^18^. Similar mechanisms exist within humans. The human counterpart of murine Vγ7+ γδ T cells expressing Vγ4+ TCR interacts with the BTNL3-BTNL8 heterodimer expressed in human intestinal epithelial cells^9^. In addition to Vγ4+ γδ T cells, there are other gut-localizing γδ T cells in humans that express Vδ2+ γδ TCR and CD103^19^. In the human intestine, CD103+Vδ2+ γδ T cells produced less cytokines when they were stimulated in vitro than their CD103− counterparts, suggesting that CD103+ γδ T cells may play immunoregulatory roles^19^.

In addition to surface molecules, various cytokines also affect the localization of γδ T cells to specific organs. Murine dermal γδ T cells utilize CCR2 and CCR6 for their localization^20^, and other γδ T cells may also utilize the chemotactic axis for their localization in target tissues. In the murine gut, intestinal epithelial cells express CCL25^21^, which chemoattracts γδ T cells expressing CCR9^22^. In addition, the gut microbiome induces CXCL-9 production;^23^ because of CXCR3+ γδ T cells^24^, the CXCL9-CXCR3 axis may also contribute to the localization of γδ T cells.

## Role of γδ T cells in mucosal protection

**Fig. 1:**
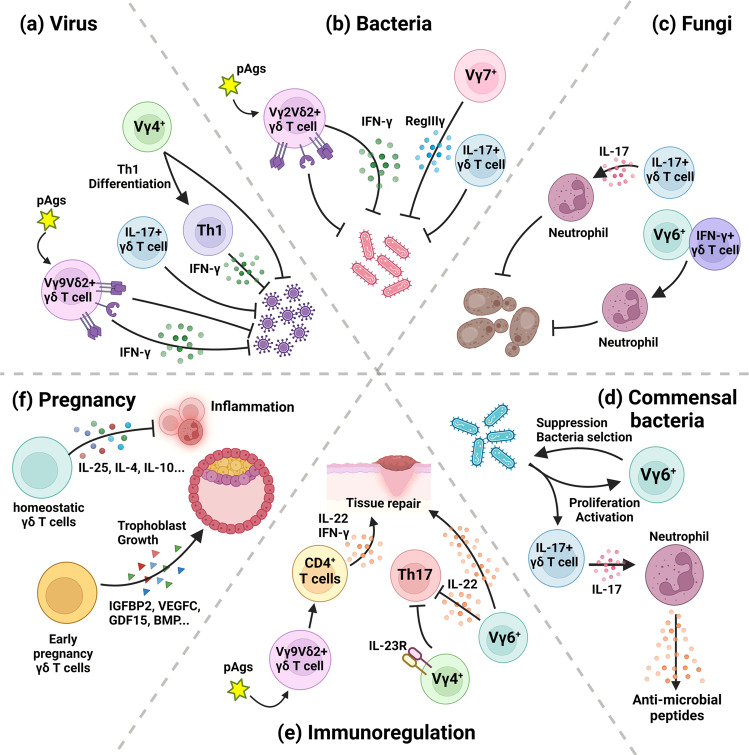
Role of γδ T cells in mucosal protection. **a** Role of γδ T cells in protection against viral infection. Murine γδ T cells protect against viral infection by producing IL-17, thereby reducing the mortality rate of influenza-infected neonates. Additionally, Vγ4+ γδ T cells upregulate IFN-γ production in CD4 T cells, thereby suppressing viral infection in vaginal HSV infection. In humans, phosphoantigen-stimulated Vγ9Vδ2 γδ T cells directly kill virus-infected cells via the Fas-FasL pathway, NKG2D activation, and the TRAIL-mediated pathway. In addition, human γδ T cells directly produce IFN-γ in the endocervix, thereby lowering the viral load. **b** Role of γδ T cells in protection against bacterial infection. IL-17-producing γδ T cells exert a protective role during early bacterial infection. In the gut, Vγ7+ intraepithelial lymphocytes (IELs) produce RegIIIγ, an antimicrobial peptide, and suppress the growth of gram-positive bacteria. In a nonhuman primate model of *M. tuberculosis* infection, Vγ2Vδ2+ γδ T cells produced IFN-γ, thereby suppressing IL-22-producing T cells and facilitating granulomas via the IFN-γ-associated axis. **c** Role of γδ T cells in protection against fungal infection. In the mouse lung, IL-17-producing γδ T cells recruit neutrophils, thereby facilitating the clearance of *Candida albicans*. Likewise, in the mouse vagina, both IL-17-producing Vγ6+ γδ T cells and IFN-γ-producing γδ T cells suppress *C. albicans* by recruiting neutrophils. **d** Role of γδ T cells in the selection of microbiota. In the mouse oral cavity, Vγ6+ γδ T cells are localized close to the biofilm and produce IL-17, thereby suppressing the outgrowth of bacteria. Since the absence of bacteria reduces the Vγ6+ γδ T-cell population, the microbiota induces the proliferation of Vγ6+ γδ T cells, which alters the cell population. Additionally, the microbiota facilitates IL-17-producing γδ T cells, which subsequently recruit neutrophils. Neutrophils produce antimicrobial peptides and suppress pathogen growth. **e** Role of γδ T cells in immunoregulation. Murine Vγ6^+^ γδ T cells produce IL-22, thereby facilitating tissue repair and suppressing pathogenic CD4 T cells. Additionally, Vγ4^+^ γδ T cells express high levels of the IL-23 receptor and suppress IL-17-expressing autoreactive T cells. Immunoregulatory γδ T cells are also present in humans. Phosphoantigen-activated human Vγ9Vδ2+ γδ T cells can act as immunosuppressive cells by facilitating CD4 T-cell polarization toward IL-22- and IFN-γ-producing populations. **f** Role of γδ T cells in pregnancy. In the homeostatic decidua, γδ T cells produce IL-25, IL-4, and IL-10 while downregulating IFN-γ. Therefore, decidual γδ T cells play immunosuppressive roles. During early pregnancy, decidual γδ T cells also produce growth factors such as IGFBP2, VEGFC, GDF15, and BMP1, directly facilitating the growth of trophoblasts. Loss of γδ T cells is related to recurrent abortion, indicating the importance of γδ T cells in sustaining pregnancy.

Because γδ T cells can colonize the mucosa, they may play various protective roles. These cells protect against pathogens, such as viruses^25–34^, bacteria^35–46^, and fungi^47–50^. In addition to their role in pathogenic infections, γδ T cells play roles in homeostasis. They participate in the selection of the microbiome^51–53^ and perform immune-regulatory roles^39,54–56^, thereby helping to sustain homeostasis in the organism. Finally, because they colonize the female reproductive tract, γδ T cells also impact pregnancy^57–62^. Figure 1a–e shows the functions and overall protective roles of γδ T cells in different mucosal tissues.

### Role of γδ T cells in protection against viral infection

Because the mucosa is the primary site for viral infection, it is not surprising that γδ T cells protect against viral infection (Fig. 1a). In the lung, murine Vγ6Vδ1 γδ T cells initially dominate among lung-resident γδ T cells, and the population shifts toward Vγ4+ γδ T cells over time^25^. In a murine influenza infection model, γδ T cells produced IL-17; therefore, they could potentially aid in resolving influenza infection and decrease the mortality in influenza-infected neonates^26^. The functionality of γδ T cells in lung protection has also been studied in humans. In a human influenza virus and human γδ T-cell xenograft model, aminobisphosphonate-pamidronate (PAM)-activated γδ T cells reduced disease severity and mortality caused by human seasonal H1N1 and avian H5N1 influenza virus and controlled lung inflammation and viral replication^27^. PAM-activated human Vγ9Vδ2 γδ T cells effectively killed influenza A virus-infected lung alveolar epithelial cells via NKG2D activation, the Fas-FasL pathway, and the TRAIL-mediated pathway^28^. In addition to direct killing, isopentyl-diphosphate-activated Vγ9Vδ2 γδ T cells exert a noncytolytic protective role by producing IFN-γ^29^.

In addition to the lung, the reproductive tract is a major route for viral infection, and γδ T cells also protect against viruses in the reproductive tract. In a murine vaginal herpes simplex virus (HSV) infection study, depletion of γδ T cells reduced mouse survival and increased viral titer, resulting in reduced IFN-γ production in CD4 T cells^30^. A BALB/C mouse HSV infection model revealed that Vγ4+ γδ T cells are recruited to the infected vagina^31^. Furthermore, a vaginal adenoviral infection study revealed that vaginal γδ T cells were activated after infection. In that study, the γδ T-cell number was positively correlated with the viral load, and the number of vaginal αβ T cells was not significantly altered. The expression of chemokine receptors and cytotoxic molecules is increased in γδ T cells^32^. In accordance with a murine study, a simian immunodeficiency virus infection model in rhesus macaques generated by Tuero et al. also revealed the importance of γδ T cells in viral infection in the vagina^33^. In that study, the majority of Vδ1+ or Vδ2+ γδ T cells were CD8+ or CD4−CD8−γδ T cells. A site-specific reactivity difference was observed in Vδ2+ γδ T cells; Vδ2+ γδ T cells in the endocervix had a higher percentage of IFN-γ-producing cells in the population than those in the vagina. Additionally, endocervical Vδ2 γδ T cells exhibited greater IFN-γ production than Vδ1+ γδ T cells in the same tissue. In the endocervix, the chronic viral load was negatively correlated with Vδ2+ γδ T cells^33^. Likewise, in a human study, a proportion of the γδ T-cell subset was related to vaginal dysbiosis and human immunodeficiency virus (HIV) susceptibility. Among HIV-infected women, the Nugent score, which represents bacterial vaginosis, was positively correlated with the Vδ1+ γδ T cell percentage. Among healthy women, however, the Nugent score was negatively correlated with the Vδ1+ γδ T cell percentage. Vδ2+ γδ T cells showed an enrichment signature that was distinct from that of Vδ1+ γδ T cells; in non-HIV-infected women, the frequency of Vδ2+ γδ T cells was correlated with vaginal dysbiosis. In contrast, in HIV-infected women, the frequency of Vδ2+ γδ T cells was not related to vaginal dysbiosis. Therefore, in HIV-negative women, bacterial vaginosis induces Vδ2+ γδ T cell accumulation and Vδ1+ γδ T cell decrement^34^.

### Role of γδ T cells in protecting against bacterial infection

The effectiveness of γδ T cells in protecting against bacteria has been studied in many organs (Fig. 1b). Lung-localized γδ T cells showed a greater activation signature than αβTCR+ T cells after *Streptococcus pneumoniae* infection in mice. Because γδ T cell expansion is confined to lung tissue, the expanded γδ T cells are tissue-resident γδ T cells^35^. The effector function of lung γδ T cells seems to be related to cytokine secretion, especially secretion of IL-17. After *Mycobacterium bovis* bacillus Calmette-Guérin (BCG) inoculation, IL-17A produced by Vγ4 or Vγ6+ γδ T cells was required for granuloma formation^36^. Likewise, during *Bordetella pertussis* infection, γδ T cells played a protective role in both an innate and an adaptive manner. During early infection of the lung, Vγ4−Vγ1− γδ T cells produce IL-17, thereby facilitating bacterial clearance. Meanwhile, Vγ4+ γδ T cells arose 7–14 days after immunization with heat-killed *B. pertussis*, and adaptive-like γδ T cells produced an increased amount of IL-17^37^. Murine CD1d presents lipid molecules produced by the commensal microbiome to IL-17+ γδ T cells, and the depletion of the commensal microbiome by treatment with antibiotics reduces the number of hepatic γδ T cells^38^. The liver γδ T-cell population is mainly composed of IL-17-producing Vγ4+ and Vγ4-Vγ1- γδ T cells, which are also found in many different organs, including the lung, during homeostatic and infectious conditions^36,39^. Therefore, CD1d-mediated lipid presentation may be a critical source of stimulation to those IL-17-producing γδ T cells for their activation and the subsequent suppression of bacteria.

The importance of lung γδ T cells was also studied in a nonhuman primate *M. tuberculosis* infection model. In the model, Vγ2Vδ2 γδ T cells were activated by (E)-4-hydroxy-3-methyl-but-2-enyl pyrophosphate (HMBPP) and produced IFN-γ, which downregulated IL-22-producing T cells that form lung granulomas via the IFN-γ-associated cytokine network^40^.

In addition to the lung, the urinary tract is also susceptible to bacterial infection, and the role of γδ T cells in the protection of the urinary mucosa has been studied by several groups. In an experimental model of urinary tract infection with *Escherichia coli*, Matsukawa et al. showed that γδ T cells infiltrated the bladder and kidney, resulting in a time-dependent increase in the number of γδ T cells^41^. The same experimental model was used by Sivick et al. and the importance of IL-17 in the acute clearance of pathogens was demonstrated^42^. IL-17 expression in the bladder was significantly decreased in γδTCR knockout (KO) mice. In addition, γδ T cells were the only T cells that had significantly upregulated expression of IL-17 after 48 h of infection, thereby demonstrating that the major source of early IL-17 in the bladder is γδ T cells^42^.

Finally, in the gut, intestinal intraepithelial lymphocytes (IELs) produce RegIIIγ, an antimicrobial peptide that kills gram-positive bacteria when pathogens penetrate the mucosal barrier. Its production requires MyD88 expression in epithelial cells, suggesting that epithelial cells act as alarm cells that send activation cues to IELs^23^. IL-15 production in intestinal epithelial cells depends on MyD88^43^. IL-15 signaling maintains γδTCR+ IELs^43^ and activates T cells even when T cells are not receiving costimulatory signals^44^. In addition, IL-15 signaling can induce the expression of NKG2D^45^. Because the gut microbiota does not affect BTNL1 or BTNL6 expression in mouse intestinal epithelial cells^46^, the response of IELs against pathogens may be independent of TCR signaling and dependent on IL-15 production in intestinal epithelial cells.

### Role of γδ T cells in protection against fungal infection

In addition to the bacterial microbiome, symbiotic fungal microbiomes exist in the mucosa, which are typically not pathogenic in healthy individuals^47^. However, in individuals with weak or compromised immune systems, such as infants or HIV patients, fungal infection can be life-threatening. Therefore, many studies on the immune responses against these symbiotic and pathogenic fungal microbiomes have been performed. The results revealed the complex roles of γδ T cells in interacting with fungal microorganisms on different mucosal surfaces (Fig. 1c). During *Candida albicans* infection in the mouse lung, a lack of γδ T cells, the major source of IL-17A in the lung, reduced neutrophil infiltration, thereby decreasing pathogen clearance^48^. In addition to the lung, a vaginal *C. albicans* infection model showed an increase in fungal colonization in mice lacking γδ T cells^49^. Likewise, both IL-17-producing Vγ6Vδ1+ γδ T cells and IFN-γ-producing non-Vγ6+ γδ T cells are present in the murine uterus, and depletion of γδ T cells increased the susceptibility of mice to vaginal *C. albicans* infection by decreasing neutrophil recruitment^50^. These results suggest that γδ T cells play a protective role in fungal infection by interacting with other immune cells, such as neutrophils.

### Role of γδ T cells in selection of the microbiota

The presence of the microbiota is critical for sustaining host homeostasis. In addition to colonizing the mucosa and preventing pathogen growth, the microbiota also affects the global status of the host. The microbiota affects autoreactive immune responses and neurological disorders and even produces crucial metabolites such as vitamins. Therefore, the selection of beneficial microbiomes is critical to sustain host health. Because γδ T cells colonize the mucosa, it is not surprising that they play important roles in microbiota selection (Fig. 1d). The role of γδ T cells in selecting organisms in the microbiota has been studied for various mucosal surfaces, such as the oral cavity, gut, and conjunctiva, using various mouse models. In the gingiva, aggressive periodontitis is associated with the outgrowth of *Aggregatibacter*, a commensal microbiome organism that dwells in the oral cavity. When γδ T cells are absent, *Aggregatibacter* shows outgrowth, demonstrating that bacterial regulation is at least partially performed by γδ T cells. Wilharm et al. also revealed the interplay between oral γδ T cells and the microbiome^51^. In the mouse gingiva, the major γδ T-cell population is Vγ6+ γδ T cells, which produce IL-17 and are localized close to the biofilm. The number and activation signatures of Vγ6+ γδ T cells are decreased in germ-free mice. This decrease in Vγ6+ γδ T cells results in increased gingival inflammation and changes in microbiota diversity^51^. As shown by Wilharm et al. the interaction of the microbiota and γδ T cells is bidirectional. In a murine bacterial pneumonia model induced by *Pseudomonas aeruginosa*, disruption of the gut flora by antibiotic treatment reduced the number of IL-17-producing γδ T cells and their cytokine production, resulting in decreased neutrophil recruitment. Because anti-γδTCR antibody treatment also produced the same symptom, the presence of commensal microbiota is considered to protect the host from pneumonia by facilitating IL-17-producing γδ T cells^52^. Likewise, in the murine conjunctiva, the commensal bacterium *Corynebacterium mastitidis* regulates IL-17-producing Vγ4+ γδ T cells. Disruption of this commensal bacterium reduced neutrophil recruitment to the conjunctiva, resulting in a decrease in the antimicrobial peptide concentration in tears. As a result, this dysbiosis increased susceptibility to *C. albicans* and *P. aeruginosa*^53^.

### Role of γδ T cells in immunoregulation

The functionality of γδ T cells is not confined to proinflammatory, antipathogenic, and cytotoxic functions. Rather, a subset of γδ T cells play an immunoregulatory role (Fig. 1e). During chronic exposure to *Bacillus subtilis*, administration of IL-22 reduced lung inflammation and subsequent fibrosis by reducing CXCR3 expression in CD4 T cells and CXCL9 expression in the lung. The major IL-22-producing cell population in the lung is Vγ6Vδ1+ γδ T cells, and knockout of γδ T cells increased pathogenic CD4 T-cell infiltration of the lung^54^. However, these IL-22-producing regulatory γδ T cells are not confined to the lung; they are also found in the mouse conjunctiva and aid in epithelial regeneration^55^. Liang et al. demonstrated that immunoregulatory γδ T cells are related to the activation state of γδ T cells^39^. Weakly activated mouse γδ T cells express IL-23R, while naïve or highly activated mouse γδ T cells do not express this receptor. IL-23R+ γδ T cells show a strong suppressive effect on IL-17+ autoreactive T cells. Because anti-IL-23R antibodies or excessive IL-23 treatment abates the suppressive effect of IL-23R+ γδ T cells, they function as an IL-23 sink. The expression level of IL-23R in immunized mice is higher in Vγ4+ γδ T cells than in Vγ1+ γδ T cells, indicating that Vγ4+ γδ T cells can act as suppressive γδ T cells^39^.

In humans, HMBPP-stimulated Vγ9+Vδ2+ γδ T cells can also act as immunoregulators by polarizing CD4 T-cell population toward IFN-γ- and IL-22-producing cells. This IL-22 skewing potential is further enhanced when Vγ9Vδ2 γδ T cells are stimulated with IL-15 rather than IL-2. This skewing ability is also related to tumor necrosis factor (TNF)-α and inducible costimulatory molecular ligand (ICOSL) because treatment with blocking antibodies reduced IL-22 production^56^.

### Role of γδ T cells in pregnancy

During pregnancy, there are dynamic proportion changes in immune cells. At the first trimester, NK cells become enriched and promote embryo development by secreting various cytokines, as well as growth factors^57^. In addition to NK cells, which are robustly studied due to their abundance and various effector functions, γδ T cells, which are present in the endometrium throughout pregnancy^58^, also affect pregnancy. The relationship between pregnancy and γδ T cells has been robustly studied in females with recurrent abortion (Fig. 1f). Under homeostatic conditions, decidual γδ T cells express IL-25 and IL-17RB, and the addition of IL-25 to decidual γδ T cells upregulates IL-4, IL-10, and Ki-67 expression and downregulates IFN-γ expression. Therefore, decidual γδ T cells play immunosuppressive roles^59^. During early pregnancy, activated and terminally differentiated proinflammatory γδ T cells are enriched in the decidua, while no significant changes are found in the blood. The enriched γδ T cells show polyclonal TCR δ or γ usage^60^. These decidual γδ T cells not only protect the decidua from infection but also regulate fetal growth. During early pregnancy, decidual γδ T cells show activated phenotypes. They express high levels of NKG2D, CD38, CD31, and HLA-DR and produce high levels of proinflammatory cytokines such as TNF-α, IFN-γ, and IL-17a. However, they show remarkably low cytotoxicity. Decidual γδ T cells also produce growth factors such as IGFBP2, VEGFC, GDF15, and BMP1, and the expression levels of these growth factors are significantly decreased in patients with recurrent abortion. These growth factor-producing γδ T cells have been shown to promote the migration, invasion, and proliferation of trophoblasts^61^.

In addition to the human study, a murine recurrent spontaneous abortion model revealed the complex and time-dependent role of γδ T cells in the decidua and pregnancy. Clark, D. A. & Croitoru, K. demonstrated that TGF-β2 and IL-10-producing γδ T cells accumulate in the decidua on Day 8.5 of gestation and express Vγ1. Depletion of γδ T cells facilitated abortion, and anti-TGF-β2/3 or anti-IL-10 antibodies augmented the abortion rate^62^.

## Role of γδ T cells in the disruption of mucosal homeostasis

**Fig. 2:**
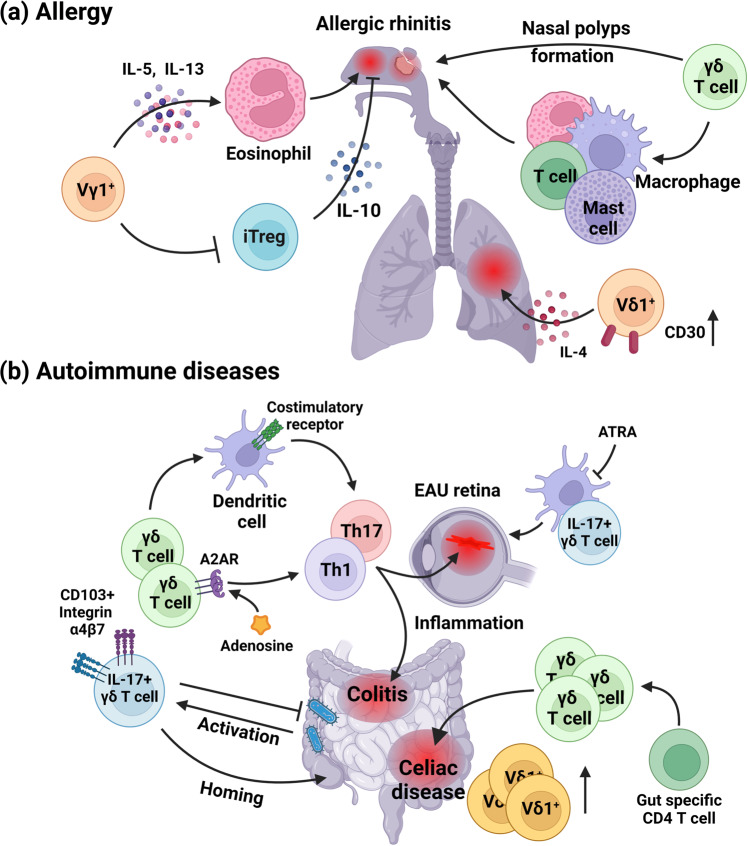
Role of γδ T cells in the disruption of mucosal homeostasis. **a** Role of γδ T cells in allergies. In mice, IL-17+ γδ T cells are related to the inflammatory symptoms of allergic rhinitis, and suppressing these cells alleviates the symptoms. Additionally, murine Vγ1+ γδ T cells expressing IL-13 and IL-5 recruit eosinophils and reduce regulatory T cells, thereby facilitating inflammation and worsening allergic responses. Consistent with the murine data, γδ T cells were significantly upregulated in the nasal mucosa of allergic rhinitis patients. These allergic γδ T cells recruit other immune cells, such as B cells, conventional T cells, macrophages, mast cells and eosinophils, and induce nasal polyp formation, which is a signature of more severe symptoms. Finally, in the human lung, Vδ1+ γδ T cells express CD30 and IL-4. These Th2-like Vδ1+ γδ T cells worsen allergy symptoms. **b** Role of γδ T cells in autoimmune diseases. In the murine experimental autoimmune uveitis (EAU) model, γδ T cells affected the maturation of dendritic cells (DCs), which subsequently induced Th1 and Th17 differentiation. Administration of retinoic acids reduced the activation of both DCs and γδ T cells, thereby alleviating inflammation. Meanwhile, adenosine, which downregulates proinflammatory responses in various cells, such as T cells, neutrophils, and macrophages, activated γδ T cells that induce Th17 differentiation. These adenosine-activated γδ T cells worsened the symptoms in the EAU model. In the gut, both murine and human γδ T cells are major players in autoimmune diseases. In mice, IL-17-producing γδ T cells induce Th17 differentiation and subsequently initiate colitis. Colitogenic γδ T cells express high levels of CD103 and integrin α4β7, indicating that these cells show high gut-homing potential. In addition to IL-17-producing γδ T cells, colon-localized γδ T cells producing IFN-γ are associated with the onset and progression of colitis, suggesting that not only IL-17-producing but also IFN-γ-producing γδ T cells in the gut can be autoreactive. These autoreactive γδ T cells are activated by the gut microbiome, since germ-free mice showed alleviated inflammation in γδ T-cell-mediated colitis. Likewise, in the human gut, upregulation of gut epithelial γδ T cells is the hallmark of celiac disease. In celiac disease patients, Vδ1+ γδ T cells are increased. Those pathogenic γδ T cells are not necessarily specific to the gluten antigen; gut-specific CD4 T cells may activate γδ T cells, which subsequently increase inflammation and worsen the symptoms of celiac disease.

Because of their efficient cytotoxic and cytokine production abilities, γδ T cells comprise the first-line defense system of mucosal surfaces. However, their strong effector function and low threshold for activation^63^ also act as risk factors for the disruption of mucosal homeostasis. Pathogenic γδ T cells induce allergies^64–69^ and autoimmune diseases^70–85^. Figure 2a, b shows the overall pathologic roles of γδ T cells in the mucosa.

### Role of γδ T cells in allergy

Because of their high level of localization in the lung and pulmonary mucosa, the role of γδ T cells in allergic responses has been robustly studied in airway allergy models and patients with allergic rhinitis (Fig. 2a). In mice, suppression of IL-17+ γδ T cells by adoptive transfer of bone marrow mesenchymal stem cells alleviated the inflammatory symptoms of allergic rhinitis^64^. In ovalbumin-sensitized and challenged mice, the Vγ1+ γδ T-cell population upregulated Th2 cytokines, such as IL-13 and IL-5, and the infiltration of eosinophils, thereby facilitating the allergic response^65^. In this paper, specific depletion of Vγ1+ γδ T cells or knockout of total γδ T cells (TCRδ-KO) significantly reduced IL-13 and IL-5 levels in bronchoalveolar lavage (BAL) fluid and eosinophil infiltration in the ova-sensitized airway; meanwhile, adoptive transfer of Vγ1+ γδ T cells to TCRδ-KO mice led to recovery of the phenotype. Since BAL fluidic IL-13 and IL-5 are nearly undetectable and immune cell infiltration into the airway after OVA sensitization is greatly decreased in TCRδ-KO mice, γδ T cells act as major drivers of allergic responses. Vγ1+ γδ T cells not only secrete Th2 cytokines and induce eosinophil infiltration but also enhance allergic responses by reducing the pulmonary accumulation of IL-10-producing CD4+CD25+ T cells^66^. In this study, depletion of Vγ1+ γδ T cells significantly upregulated the IL-10-producing cell population among pulmonary cells. Depletion of Vγ1+ γδ T cells increased the number of both Foxp3+ cells and FR4+ cells, which represent regulatory T cells (Tregs) and antigen specific Tregs, respectively. Therefore, Vγ1+ γδ T cells globally suppress the immunosuppressive Treg population, thereby enhancing allergic responses^66^.

Consistent with the mouse model, γδ T cells were significantly increased in the nasal mucosa of allergic rhinitis patients. γδ T-cell infiltration was positively correlated with the infiltration of other immune cells, such as eosinophils, macrophages, mast cells, B cells, and conventional T cells. γδ T cells and macrophages exhibit close proximity in the mucosa, indicating cell‒cell interactions of γδ T cells with other types of immune cells^67^. Lee et al. demonstrated that the presence of γδ T cells is related to the presence of nasal polyps, which is related to severe symptoms^68^. They showed that the expression of Vγ1+ γδ T cells was higher in patients with eosinophilic chronic rhinosinusitis with nasal polyps than in those without nasal polyps, and the presence of γδ T cells was related to a higher recurrence rate and worse symptoms^68^. Finally, Vδ1+ γδ T cells in the human lung have been shown to express a Th2-like signature, with upregulation of CD30 and production of IL-4. These Th2-like Vδ1+ γδ T cells worsen allergy symptoms^69^.

### Role of γδ T cells in autoimmune diseases

Although their increased activation signature and specificity toward stress molecules make γδ T cells efficient defenders against pathogens, their phenotype can also intensify tissue damage when they perform deleterious effector functions, such as unrestrained inflammation. Therefore, γδ T cells are also major players in autoimmune diseases. The role of murine γδ T cells in autoimmune diseases has been well studied in uveitis and colitis models (Fig. 2b). In an experimental autoimmune uveitis (EAU) model induced by interphotoreceptor retinoid-binding protein, γδ T cells affected the maturation of dendritic cells (DCs), which subsequently induced the maturation of CD4 T cells into Th1 and Th17 subsets by upregulating the expression of costimulatory receptors on DCs^70^. In the article, the clinical score of EAU mice in the TCRδ-KO is significantly lower than that of control mice, which is correlated with the decrease in the number of CD11c+ DCs in the spleen. In addition, the percentage of IFN-γ+ or IL-17+CD4+ T cells was decreased in TCRδ-KO mice, indicating the importance of γδ T cells in CD4+ T-cell functionality. γδ T cells can affect CD4+ T cells both directly and indirectly; coculture of CD4+ T cells with γδ T cells upregulates the proliferation of CD4+ T cells, and proliferation was further increased when DCs were added to the culture. Therefore, γδ T cells can work as a critical factor in autoimmune responses by regulating CD4+ T-cell activation in various ways. Similar to the results of this study, Liang et al. revealed that depletion of CD25-expressing DCs reduced Th17 responses and the γδ T-cell population in an EAU model^71^. They also revealed that all-trans retinoic acid (ATRA) decreased the number of CD25+ DCs, as well as IL-17-producing γδ T-cell activation. ATRA-treated γδ T cells showed a reduced ability to induce Th17 differentiation, and EAU model mice treated with ATRA exhibited lower disease scores. Therefore, in the eye, retinoic acids may regulate γδ T cells and CD25+ DCs, which comprise a possible autoreactive and inflammatory population^71,72^.

In contrast to the straightforward action of retinoic acids, which directly suppress γδ T cells, the interplay of adenosine monophosphates (AMPs) and γδ T cells is more complex. AMP is processed into adenosine by CD73 and binds to adenosine receptor A2 (A2AR). A2AR signaling reduces IFN-γ and IL-4 production in CD4 T cells^73,74^ and expands regulatory T cells^75^. In addition, adenosine reduces neutrophils^76^ and macrophages^77^, acting as a strong immune suppressor in both the innate and adaptive immune systems. However, the action of adenosine on γδ T cells is distinct from its action on other immune cells. γδ T cells express high A2AR levels but low CD73 levels. γδ T cells act as adenosine sinks that suppress adenosine binding to other cells and become activated by A2AR signaling^78^. A2AR-activated γδ T cells enhanced Th17 differentiation^79^, thereby facilitating inflammation and worsening symptoms in an EAU model.

Similar to eyes, autoimmune diseases in the gut can also be caused by gut-resident γδ T cells (Fig. 2b). Generally, Th17 cells are considered the main cause of colitis. However, Do et al. showed that while TCRβ KO mice are highly susceptible to colitis, mice lacking both αβTCR and γδTCR showed resistance to disease onset^80^. Transfer of IL-17+ γδ T cells and CD4 T cells induced Th17 differentiation and subsequent colitis, indicating the critical role of IL-17+ γδ T cells in the onset of colitis^80^. Do et al. also revealed that pro-colitogenic γδ T cells express CD103 and integrin α4β7, indicating strong gut-homing potential. Meanwhile, in another study, γδ T cells localized in the colon and produced IFN-γ; γδ T cells also exhibited a correlation with the onset and progression of colitis, suggesting that not only IL-17-producing but also IFN-γ-producing γδ T cells in the gut can be autoreactive^81^. Because γδ T-cell-mediated colitis is alleviated under germ-free conditions, microbiome-mediated γδ T-cell activation is the major cue for the production of autoreactive γδ T cells^82^.

Finally, in humans, gut intraepithelial γδ T cells are a hallmark of celiac disease. The TCR repertoire of intraepithelial γδ T cells is highly diverse in celiac patients. In the homeostatic human gut, γδ T cells use Vδ3 as their γδTCR, while in celiac disease patients, Vδ1 utilization is increased^83^. Additionally, the inclusion of *TRDV1* and *TRDV3* becomes more frequent^84^. These celiac-associated γδ T cells are not necessarily specific to gluten antigens; Risnes et al. found that gut-specific CD4 T cells induce γδ T-cell expansion, and these expanded gut intraepithelial γδ T cells share a similar TCR repertoire with γδ T cells in the blood^85^. Therefore, even though they do not directly recognize antigens, γδ T cells may amplify symptoms in autoimmune diseases.

## Application of γδ T cells in clinical practice

### Obstacles for utilizing γδ T cells for clinical application

**Fig. 3:**
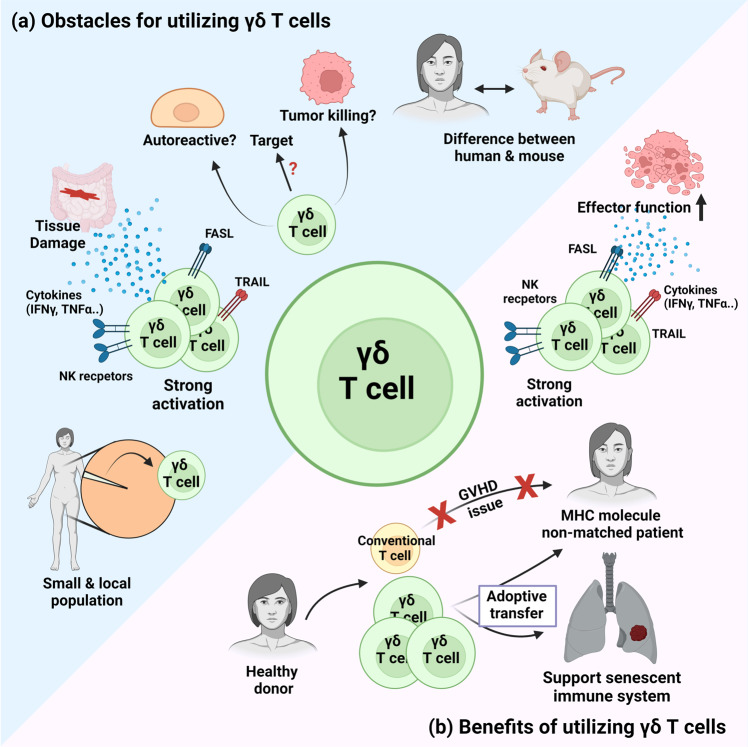
Obstacles and benefits of utilizing γδ T cells for clinical application. **a** There are several obstacles to utilizing γδ T cells in clinical practice. Due to gaps between murine and human γδ T cells, direct application of experimental results obtained from murine systems in human clinical practice is restrained. Additionally, insufficient knowledge about the targets of γδ T cells makes γδ T-cell utilization difficult since several target molecules are expressed on homeostatic tissue, and targeting those homeostatic tissues can redirect activated γδ T cells to normal tissues and induce off-target toxicity. Their small population size and tissue-localizing features are also major obstacles for γδ T-cell utilization because targeting tissue-specific γδ T cells requires specialized delivery techniques. Finally, because of the strong activation features of γδ T cells, they may have unexpectedly large effects on local tissues. **b** Several benefits of utilizing γδ T cells in clinical applications make them attractive subjects for further study. Because γδ T cells do not recognize MHC molecules, they can be transferred to MHC-mismatched recipients without inducing graft-versus-host disease (GvHD)-related issues and can be used for off-the-shelf therapy. In old adults and cancer patients, the functionality of immune cells is decreased due to immunosenescence. The adoptive transfer of γδ T cells obtained from young and healthy donors can be a solution for immunosenescence without GvHD issues. Strong effector functions of γδ T cells may also be a benefit under well-controlled conditions, since a small number of γδ T cells can fulfill the requirement of a therapeutic dose. Therefore, strong effector functions can make γδ T cells cost-effective and less time-consuming therapeutic agents.

Despite the long period since their discovery in 1986, the application of γδ T cells in human clinical trials does not have a long history. Recently, growing interest in the cytotoxic effector functions of γδ T cells has resulted in many research groups applying γδ T cells in antitumor clinical trials^86,87^. However, the application of γδ T cells for the treatment of other diseases is lacking. There are several obstacles to the application of γδ T cells in clinical trials (Fig. 3a). First, the difference between human and mouse γδ T cells increases the difficulty of applying γδ T cells for studies and conditions. Although several groups have found some commonalities between mouse and human cells^9,88^, several differences still exist between human and mouse γδ T cells. For example, there is no direct homolog of human Vγ9Vδ2 γδ T cells, which are the most common γδ T cells in human peripheral blood. Although some studies have suggested similarities between human Vγ9Vδ2 γδ T cells and murine γδ T-cell subsets^89^, these similarities are restricted to certain features and not the entire cell. Therefore, trials to fill the gap between mouse and human systems is required for adaptation of mouse studies to human clinical practice. In addition to the missing links between mice and humans, insufficient knowledge of the targets of γδ T cells is also a critical obstacle restraining the utilization of γδ T cells in clinical applications. There are some known targets of mouse and human γδ T cells^90,91^. Among them, however, R-phycoerythrin is not a naturally found protein in humans, and tRNA synthetases are intracellular proteins. Therefore, even though these proteins are known targets of γδ T cells, it is difficult to utilize them in clinical trials. In addition, other known ligands are not strictly related to pathogenic conditions and thereby can redirect activated γδ T cells to normal tissues or targets and induce off-target toxicity. Although Vγ9Vδ2 γδ T cells show specificity toward infected or transformed cells^92^, other γδ T cells can recognize MHC or MHC-like molecules without foreign antigen presentation^90^, which may induce autoreactivity.

The small population size of γδ T cells is also a major hurdle for their proper utilization. Comprising only 0.5–10% of total human peripheral blood^93^, γδ T cells are a minor population among T cells. In addition, their tissue localization makes them a difficult target for utilization because the tissue-specific delivery of drugs or the activation of cells requires specific delivery techniques^94^. Furthermore, because of their stronger activation than conventional T cells^63^, a small number of γδ T cells may have unexpectedly large effects on local tissue. Therefore, targeting γδ T cells is difficult because of the requirement for high resolution or targeting techniques and a small window for the moderate activation of γδ T cells.

### Benefits of utilizing γδ T cells for clinical application

Despite the presence of several obstacles to γδ T-cell utilization, there are still several benefits of utilizing these cells (Fig. 3b). One of the most valuable features of γδ T cells in clinical application is their plausibility for allogeneic transfer. Because γδ T cells do not recognize MHC molecules, γδ T cells obtained from healthy donors can be applied to patients without inducing graft-versus-host disease (GvHD)-related issues^95^. With this lack of alloreactivity, γδ T cells are expected to become effector cells for off-the-shelf therapy. Makkouk et al. showed that in a hepatocellular carcinoma model, human Vδ1+ γδ T cells expressing a chimeric antigen receptor (CAR) suppressed tumors without xenogeneic GvHD issues^96^. These allogeneic γδ T-cell transfer studies have been performed in human clinical conditions and have shown positive prognostic outcomes in cholangiocarcinoma^86^ and pancreatic cancer^87^. Because the mucosa is one of the most common sites of cancer^97^ and since γδ T cells have shown therapeutic effects on solid tumors^86,87^, γδ T cells can be an effective solution for mucosal cancers.

There is another benefit for off-the-shelf therapy. Immune cells gradually lose their activity and become senescent in older adults, which is called immunosenescence. As a result, immune cells, especially T cells, lose their clonality, and cytotoxic or cytokine-secreting effector functions are diminished. This immunosenescence is observed not only in aged adults but also in tumor-infiltrated immune cells^98^. In this condition, due to senescent and the lower-functioning immune cells, various immunotherapeutic procedures, such as autologous cell therapy or immune checkpoint blockade, show low effects. In this condition, transferring healthy T cells obtained from healthy and young donors can support the senescent immune system, and using γδ T cells that do not induce GvHD issues is a considerable option.

A strong effector function may also be a benefit of utilizing γδ T cells in clinical applications. Under well-controlled conditions, transfer of a small number of γδ T cells to obtain the desired effect could be cost effective and less time-consuming. The effectiveness of γδ T cells also originates from their ability to produce both innate and adaptive immune responses. In addition to their cytotoxic effector functions and cytokine secretion, which resemble conventional T-cell responses, γδ T cells can act as phagocytes^99^ and antigen-presenting cells^56,100^. Therefore, γδ T cells can manipulate adaptive immune responses, thereby having the potential to initiate robust immune responses with small cell numbers. Therefore, the multifunctional ability of γδ T cells makes them attractive targets for clinical use, and further studies on γδ T cells will make these expectations plausible.

There still remain some obstacles for allogeneic cell therapy using γδ T cells. Current techniques for obtaining and expanding tissue-specific γδ T cells are expensive and require invasive surgeries for donors. In addition, host allorejection is another major hurdle for allogeneic adoptive transfer, and there are several preclinical trials that show evasion of the immune responses from host T cells and NK cells^101–103^. Therefore, efficient expansion protocols and tissue-specific γδ T-cell-obtaining techniques, as well as additional engineering procedures to evade rejection, are needed to make the off-the-shelf transfer of γδ T cells from healthy donors to patients a plausible and effective therapeutic procedure.

## Concluding remarks

In this review, the roles of γδ T cells in the mucosa are discussed. Although they comprise a minor population, γδ T cells play major roles in both mucosal protection and destruction. Because of their effector functions, such as cytotoxicity and cytokine secretion, γδ T cells defend the mucosa from viral, bacterial, and fungal infection. They also sustain homeostasis by selecting beneficial microbiota and performing immunoregulatory roles. γδ T cells even perform regulatory roles during pregnancy by directly producing growth factors. Despite these beneficial roles, γδ T cells can also damage mucosal health. Their robust and effective effector functions make them strong amplifiers of inflammation, thereby worsening allergic responses. In addition, γδ T cells play a central role in autoimmune diseases. Because of these beneficial and detrimental roles, γδ T cells act as a double-edged sword in the mucosal immune system. Therefore, targeting γδ T cells for clinical application could be an effective treatment for diseases. Although several obstacles exist for targeting or utilizing γδ T cells in clinical practice, they have the potential to become game-changing therapeutic agents.
